# Madeiran *Arabidopsis thaliana* Reveals Ancient Long-Range Colonization and Clarifies Demography in Eurasia

**DOI:** 10.1093/molbev/msx300

**Published:** 2017-12-05

**Authors:** Andrea Fulgione, Maarten Koornneef, Fabrice Roux, Joachim Hermisson, Angela M Hancock

**Affiliations:** 1 Max F. Perutz Laboratories, University of Vienna, Vienna, Austria; 2 Vienna Graduate School of Population Genetics, Vienna, Austria; 3 Max Planck Institute for Plant Breeding Research, Cologne, Germany; 4 Wageningen University, Wageningen, The Netherlands; 5 LIPM, Université de Toulouse, INRA, CNRS, Castanet-Tolosan, France; 6 Department of Mathematics, University of Vienna, Vienna, Austria

**Keywords:** demography, *Arabidopsis thaliana*, population genetics, relict, admixture, island

## Abstract

The study of model organisms on islands may shed light on rare long-range dispersal events, uncover signatures of local evolutionary processes, and inform demographic inference on the mainland. Here, we sequenced the genomes of *Arabidopsis thaliana* samples from the oceanic island of Madeira. These samples include the most diverged worldwide, likely a result of long isolation on the island. We infer that colonization of Madeira happened between 70 and 85 ka, consistent with a propagule dispersal model (of size ≥10), or with an ecological window of opportunity. This represents a clear example of a natural long-range dispersal event in *A. thaliana*. Long-term effective population size on the island, rather than the founder effect, had the greatest impact on levels of diversity, and rates of coalescence. Our results uncover a selective sweep signature on the ancestral haplotype of a known translocation in Eurasia, as well as the possible importance of the low phosphorous availability in volcanic soils, and altitude, in shaping early adaptations to the island conditions. Madeiran genomes, sheltered from the complexities of continental demography, help illuminate ancient demographic events in Eurasia. Our data support a model in which two separate lineages of *A. thaliana*, one originating in Africa and the other from the Caucasus expanded and met in Iberia, resulting in a secondary contact zone there. Although previous studies inferred that the westward expansion of *A. thaliana* coincided with the spread of human agriculture, our results suggest that it happened much earlier (20–40 ka).

## Introduction

Evolution takes its own course on islands (Losos and Ricklefs 2009), fuelled by local forces and conditions. Often the simplicity in island demography allows the retention of cleaner genomic signatures of local demographic and adaptive processes. Oceanic islands further represent ideal systems to study long-distance seed dispersal events. Despite their critical importance in evolutionary and ecological dynamics (Darwin 1859; Carlquist 1966), our quantitative understanding of these events is still limited by available data (Cain et al. 2000; Nathan 2006). Island colonization is generally thought to be associated with strong bottlenecks (Whittaker 1998), which reduce genetic diversity and effective population sizes (Nei et al. 1975). Moreover, islands usually have a smaller geographic extent, and consequently lower carrying capacity compared with mainlands (MacArthur and Wilson 1967), which may again reduce long-term effective population sizes, as well as the efficiency of selection. However, empirical support for these hypotheses remains inconclusive (Johnson and Seger 2001; Woolfit and Bromham 2005; Wright et al. 2009; James et al. 2016). Finally, islands may open a window to the past, as exemplified in humans, where the ∼5,000-year-old Tyrolean Iceman shows most recent relatedness with present-day inhabitants of the island of Sardinia (Keller et al. 2012). The relative isolation of island populations provides shelter from the complexities of continental demography, retaining variation that is quickly reshuffled on the continents.

*Arabidopsis thaliana* is an excellent model for investigating evolution on islands and reconstructing the dynamics of relationships to continental populations. The availability of an extensive worldwide data set allows one to reliably infer the origin and history of island clades. Further, the rigorous genome annotation supports a functional understanding of adaptive processes, which bear the potential to leave clearer genomic signatures in island populations. Indeed, single accessions from Macaronesian islands, divergent at the genomic and phenotypic levels, have been the focus of several key studies of Arabidopsis genetics (Alonso-Blanco, El-Assal, et al. 1998; Alonso-Blanco, Peeters, et al. 1998; Alonso-Blanco et al. 1999, 2003). However, the study of island *A. thaliana* at the population level has been largely neglected so far. Although the collection of natural accessions is exceptional relative to the resources available in other plant species, it only includes single samples from oceanic islands (1001 Genomes Consortium 2016). Given that the discovery of relict clades on the Iberian peninsula (1001 Genomes Consortium 2016) and in Africa (Durvasula et al. 2017) has been a key finding in recent population genomic studies, an in-depth analysis of oceanic islands off the Iberian and African coasts seems to be particularly promising.

Here, we sequenced novel samples of *A. thaliana* from the oceanic island of Madeira. We reconstructed the details of the long-distance dispersal event that accompanied colonization, as well as the demographic and adaptive history of this island clade. Moreover, Madeiran samples provide new insights into the history of continental clades, informing a new estimate for timing of major migration events in Eurasia, as well as revealing the origin and recent admixture history of the Iberian relicts, a particularly interesting and diverged clade in Eurasia (1001 Genomes Consortium 2016).

## Results

**Fig. 1.**
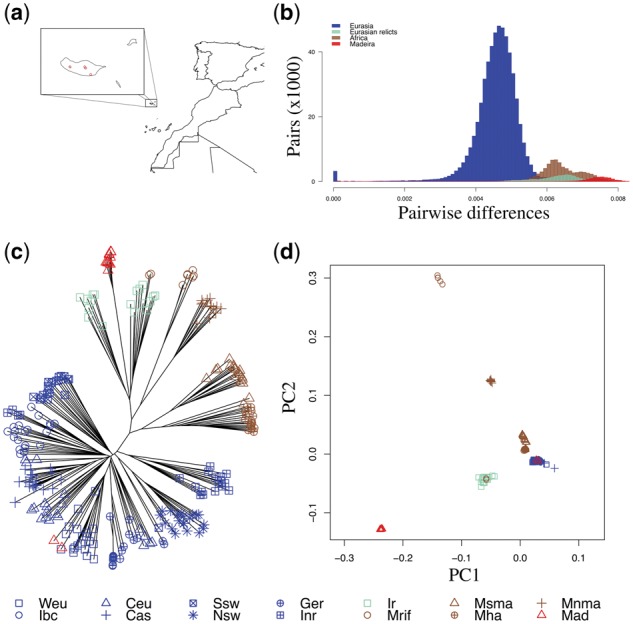
Population structure for Madeiran and worldwide samples. (*a*) Map of Madeiran samples. (*b*) Distribution of pairwise differences per base pair. (*c*) Neighbor joining tree of worldwide and Madeiran samples. (*d*) Principal component analysis of worldwide and Madeiran samples. Weu: Western Europe; Ibc: Italy, Balcans, Caucasus; Ceu: Central Europe; Cas: Central Asia; Ssw: South Sweden; Nsw: North Sweden; Ger: Germany; Inr: Iberian nonrelicts; Ir: Iberian relicts; Mrif: Moroccan Rif; Msma: Morocco South Middle Atlas; Mha: Morocco High Atlas; Mnma: Morocco North Middle Atlas; Mad: Madeira.

We sequenced the genomes of 14 accessions from the volcanic island of Madeira and analyzed these together with >1,200 samples from previous publications (1001 Genomes Consortium 2016; Durvasula et al. 2017). Our collection includes two major mountain peaks (Pico do Areeiro and Pico Ruivo) and a third site (Rabacal) in the Madeira Natural Park (11 samples), as well as samples from the Palheiro public gardens, which were growing at much lower altitude and with high levels human disturbance (three samples) (fig. 1*a* and supplementary table S1, Supplementary Material online). To represent worldwide *A. thaliana* for comparison, we used 1,135 Eurasian and 79 North African samples. Previous studies identified nine Eurasian clusters (1001 Genomes Consortium 2016), of which eight belong to the major Eurasian clade and one diverged group of samples belongs to a clade from the Iberian peninsula (the Iberian relicts), and four North African clusters (Durvasula et al. 2017). To avoid biases due to uneven sample sizes, we evenly subsampled the 13 mainland clades in our analyses, and resampled across the larger set to estimate confidence in the results (see Materials and Methods).

### Origin of Madeiran *A. thaliana*

In order to put Madeiran samples in the context of worldwide variation, we computed pairwise differences across all pairs of samples (fig. 1*b*). Strikingly, Madeirans from the Madeira Natural Park are more diverged from other worldwide samples ([0.75 ± 0.03]% differences per base pair) than all previously identified relict populations (divergence per base pair of Eurasian relicts from other mainland clades: [0.62 ± 0.06]%, and of Africans: [0.64 ± 0.05]%). In addition, diversity within Madeirans is reduced 4.3-fold compared with species-wide levels ($\theta_{\pi }$ in Madeira = 0.112%, $\theta_{\pi }$ worldwide = 0.483%).

**Fig. 2.**
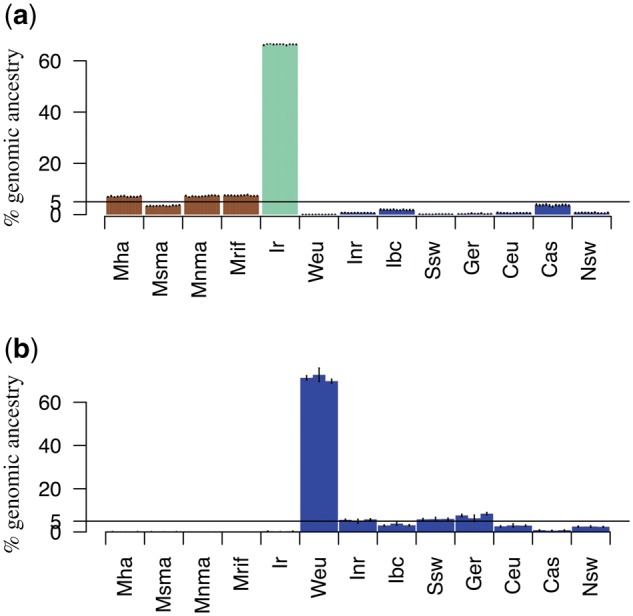
ChromoPainter analysis of Madeiran samples. (*a*) ChromoPainter analysis of the 11 native madeirans. Columns represent the percentage of Madeiran genomes that first coalesces with members of each potential donor group. Eurasian groups are colored in blue, Iberian relicts in green, and African groups in brown. The IDs for all mainland clades are described in figure 1. Vertical lines represent confidence intervals (± 1.96 · SE) across replicated analysis. (*b*) ChromoPainter analysis of the three recent migrants.

To understand the origin of Madeiran *A. thaliana*, we analyzed population structure worldwide using several complementary approaches. Based on the neighbor joining algorithm, the majority of Madeirans (11 accessions, henceforth, the native Madeirans) are nested into the Iberian relicts clade, and separated from this group by a long branch, reflecting a long divergence time. Conversely, the three samples collected in the Palheiro public gardens (supplementary table S1, Supplementary Material online), cluster with Eurasian nonrelicts. Close similarity with Western European samples suggests that these samples were recently introduced to the island (fig. 1*c*). A principal component analyses of worldwide samples corroborates these results, highlighting the strong signal of genome-wide divergence of native Madeirans to the mainland clades, with closest relatedness to Iberian relicts (fig. 1*d*). Since relatedness can vary across the genome, we further explored relatedness at the level of gene genealogies. For this, we applied the HMM-based approach ChromoPainter (Lawson et al. 2012), using the 13 mainland clades as potential donor populations. The Iberian relicts are by far the closest relatives to the 11 native Madeirans (supported, on an average, by 66.5% of their genomes, fig. 2*a*), whereas the three recent migrants clearly immigrated recently from western Europe (fig. 2*b*). Signals in other potential source populations are low and likely due to stochasticity in the inference and incomplete lineage sorting. The results for both Madeiran clusters are highly consistent across subsamples of the data set and across replicated analyses (see Materials and Methods). The two Madeiran clusters show nearly zero relatedness with the donor population that constitutes the major signal in the other group (<1% W. Eur. for the 11 native Madeirans, <1% Iberian relicts for the three recent migrants). Although coexisting on the same island, the two clades are found in different habitats, and there is no evidence they exchanged any genetic material.

Taken together, our results show that native Madeirans represent the strongest signal of divergence in the worldwide collection of *A. thaliana* samples known today, likely due to longer isolation of these island samples compared with mainland relicts. Further, our results imply that Madeiran *A. thaliana* originated from an ancestral population in common with the Iberian relicts, with the exception of three very recent migrants.

### Demographic History of *A. thaliana* in Madeira

To infer the demographic history of native Madeiran *A. thaliana*, we used the multiple sequentially Markovian coalescent MSMC (Schiffels and Durbin 2014). First, we inferred effective population size as a function of time, $N_{e}(t)$, in Madeira and Iberian relicts. Then, we inferred split times based on the cross coalescence rate (*CCR*), defined as the ratio of coalescence rates between groups to the average coalescence rate within groups across time intervals. In the time frame when the ancestral population remains uniform, the *CCR* statistic is expected to fluctuate around one, and as different clades split from each other, *CCR* decays to zero. Finally, we repeated our inference with the composite-likelihood approach *δ*a*δ*i (Gutenkunst et al. 2009).

**Fig. 3.**
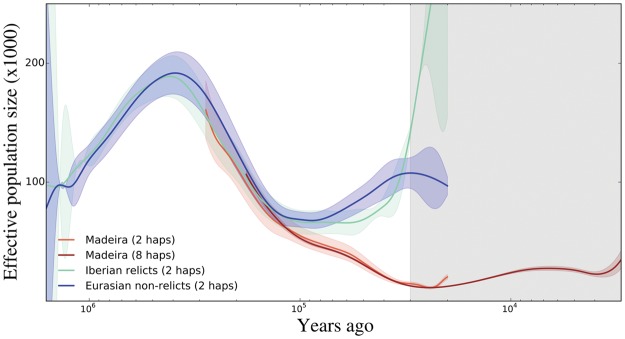
Effective population size as a function of time ($N_{e}(t)$) in Madeira (two shades of red), Iberian relicts (green) and Eurasians (blue). Inference from MSMC is shown for Madeira in two- and eight-haplotype mode, respectively, in the time frame between 20 and 300 ka, and between 3 and 200 ka. Madeiran *N_e_* is not shown at more ancient times because of inference limitations in small populations (see Materials and Methods, and supplementary fig. S2, Supplementary Material online). Inference from MSMC for Iberian relicts and Eurasians is shown between 20 ka and 1.6 Ma. Iberian relicts represent also the ancestral population in common with Madeira, before ∼70–85 ka. $N_{e}(t)$ is shown smoothed with a cubic spline across median *N_e_* for each time segment used in MSMC. Shaded areas represent confidence intervals (± 1.96 · SE). The gray area shades inference in two-haplotype mode that are less reliable in the recent past (Schiffels and Durbin 2014).

**Fig. 4.**
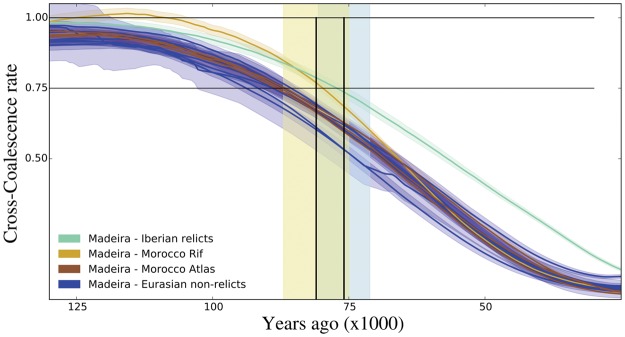
Split time between Madeira and Iberian relicts. For comparison are shown also split times between Madeira and all other mainland groups. *CCR* changes across time are shown smoothed with a cubic spline across median *CCR* for each time segment used in MSMC. Shaded areas represent confidence intervals (± 1.96 · SE). *CCR* decay is expected to cross the real split time between 0.70 <*CCR* <0.85 (see Materials and Methods). The vertical line at 75.9 ka, and the area in light blue represent the expectation and range of split times between Madeira and Iberian relicts, based on *CCR*. The vertical line at 81.0 ka, and the area in khaki represent the expectation and range of the same split time, based on *δ*a*δ*i.

Effective population size (*N_e_*) in Iberian relicts aligns with that of Eurasia in more ancient times than ∼120 ka (fig. 3). At around 40 ka, there is a spike in *N_e_* in Iberian relicts, which may be caused by population structure or admixture, as well as by an increase in census population size. *N_e_* trajectories of Madeirans and Iberian relicts start to diverge at ∼80 ka, consistent with a population split around this time. MSMC does not capture any signature of a strong colonization bottleneck. Long-term effective population size in Madeira (fluctuating around *N_e_*=30 K) is markedly reduced compared with that on the continents, consistent with a lower carrying capacity in a small oceanic island. Between 10 and 40 ka, *N_e_* in Madeira drops to even lower values (around *N_e_* = 10 K). *N_e_* for Madeiran samples is shown starting at 300 ka because of limitations of the MSMC inference in small populations (see Materials and Methods, and supplementary fig. S2, Supplementary Material online, for results across the full range of time). Consistent with our analyses of population structure, Madeirans split most recently from Iberian relicts, between 70 and 80 ka based on *CCR* (fig. 4, see Materials and Methods for the *CCR* threshold used).

Since MSMC has been shown to smooth instantaneous events across time, which affects the precision of results, it can be difficult to interpret specific timings and magnitudes of changes in $N_{e}(t)$ (Schiffels and Durbin 2014). Therefore, we examined specific features of our inference with a simulation scheme. We simulated genome-wide data under alternative demographic scenarios with the coalescence simulator msprime (Kelleher et al. 2016), and inferred $N_{e}(t)$ on the simulated data using MSMC. We then compared the $N_{e}(t)$ estimates from the simulated data sets to the one estimated from real data (see Materials and Methods for details). In particular, we used this procedure to explore 1) the split time between Madeira and Iberian relicts, 2) the population size at colonization, 3) the presence or absence of secondary gene flow, and 4) the long-term demography on Madeira (sketches of all demographic scenarios explored are shown in supplementary figs. S3, S4, and S6–S8, Supplementary Material online).

Comparisons with simulated data illustrated that the slope of decay of *CCR* between Madeirans and Iberian relicts is shallower than expected with an instantaneous population split (supplementary fig. S3, Supplementary Material online). The usual interpretation for this pattern would be gene flow after colonization, or a secondary colonization event (Schiffels and Durbin 2014). However, any other factor increasing variance in CCR may underlie this pattern, for example, variation in mutation rate across the genome. Indeed, simulations that account for an increased variance of the mutational process produce a very shallow decay in *CCR*, compatible with real data, without any migration after a split (supplementary fig. S4, Supplementary Material online). We also simulated data under models with multiple colonization events, but none of these fit the data well (supplementary fig. S4, Supplementary Material online). Overall, considering that variation in mutation rates across the genome is clearly a real phenomenon (Benzer 1961; Hodgkinson and Eyre-Walker 2011), whereas continuous long-range dispersal events are unlikely, a single introduction to the island is the most parsimonious scenario consistent with data.

Our inference with MSMC produced a baseline demographic model following inferred $N_{e}(t)$ in Madeira between present and 200 ka, and in Iberian relicts, representing the parent population of Madeirans, for times more ancient than 200 ka (supplementary fig. S5, Supplementary Material online). We next asked whether changes in the demography from the baseline model lead to observable changes in the estimated *N_e_*. In particular, we used simulations to determine the effect a colonization bottleneck would have on MSMC results (see Materials and Methods). The observed $N_{e}(t)$ are inconsistent with a strong colonization bottleneck (severity >0.1, where severity = bottleneck duration/bottleneck size; Tajima 1989), which would be visible in the MSMC framework (supplementary fig. S6, Supplementary Material online). Rather, no bottleneck, or at most a weak one (severity ≤0.1) might have accompanied the colonization of Madeira. We also investigated whether the minimum in *N_e_* between 10 and 40 ka may represent the colonization of Madeira, rather than the ice age, smoothed across time in MSMC. Simulations with a sharp colonization bottleneck in this time frame, even with duration up to 500 years, could not recover the broad signal in real data (supplementary fig. S7*a* and *b*, Supplementary Material online).

For a test of our demographic inference using a different set of summary statistics (namely the joint site frequency spectrum), we applied *δ*a*δ*i (Gutenkunst et al. 2009) to our data. The results are consistent with MSMC, in particular inference from *δ*a*δ*i produced largely overlapping estimates of the split time, varying between 75 and 87 ka across a range of demographic models (supplementary table S2, Supplementary Material online). Moreover, long-term *N_e_* in Madeira was roughly consistent with MSMC, as well as the absence of signature of a colonization bottleneck (see Supplementary Material online).

In conclusion, our inference supports a single split between Madeiran *A. thaliana* and the Iberian relicts ∼70–87 ka, with no evidence of later contact with any mainland clade, and no strong bottleneck at colonization. In addition to the baseline model, a range of similar scenarios are broadly consistent with the data. A model following $N_{e}(t)$ inference until colonization of Madeira, then stabilizing at carrying capacity (*N_e_* = 30 K) besides a long ice age bottleneck (*N_e_* = 10 K between 15 and 40 ka) with either a smooth or sudden recovery of population size to carrying capacity matched real data equally well. Including the possibility of a mild colonization bottleneck (severity = 0.1), or no bottleneck, a total of six alternative candidate demographic models are consistent with the data (supplementary fig. S8, Supplementary Material online).

### Adaptation in Madeiran *A. thaliana*

**Fig. 5.**
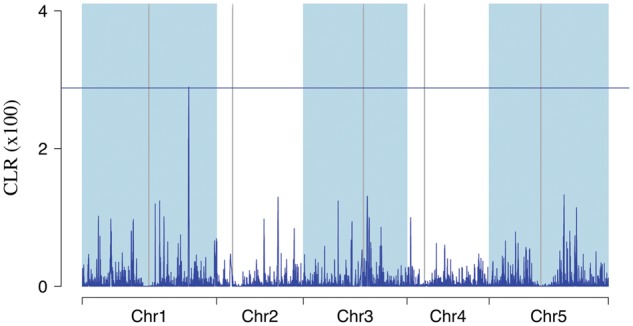
CLR in Madeiran *A. thaliana*. *CLR* statistic computed with SweepFinder2 (DeGiorgio et al. 2016), using only polymorphic sites. The horizontal line represents a significance threshold obtained with simulations assuming the best fitting demographic models (see Materials and Methods, and supplementary fig. S8, Supplementary Material online).

We scanned native Madeiran genomes for signatures of selective sweeps with sweepFinder2 (DeGiorgio et al. 2016). The significance threshold was determined with simulations (see Materials and Methods), assuming the six best fitting demographic models (supplementary fig. S8, Supplementary Material online). One single genomic region had a significant signature of selection in both sweepFinder2 configurations (fig. 5 and supplementary fig. S9, Supplementary Material online). This main peak in CLR overlaps with a region previously shown to carry the major signal of selection in Eurasian *A. thaliana* (Clark et al. 2007; Long et al. 2013). Interestingly, in this case the Madeiran samples carry the ancestral haplotype rather than the derived translocation, which was to our knowledge not yet associated with selection signatures.

To detect early adaptations to the island conditions, we used a comparative approach, the McDonald–Kreitman test (McDonald and Kreitman 1991). Of the 27,655 genes in *A. thaliana*, 11,412 had enough information to perform the test (all columns and rows in the contingency table nonempty). Of these, 48 produced a *P* value <0.01, of which 15 had a positive alpha, consistent with positive selection (see Supplementary Material online). These included three genes related to pathogen resistance (*AT2G28960*, *AT4G19050*, *AT5G11250*), as well as two Pentatricopeptide repeat genes (PPR, *AT5G06540* and *AT5G42310*) associated with chloroplast function and a candidate gene required for nucleotide excision repair and damage tolerance to UV radiation (*AT4G17020*, Kunz et al. 2005). Notably, response to pathogens and light have been suggested as biological processes underlying adaptation to altitude in natural populations of *A. thaliana* (Gunther et al. 2016), and all native Madeirans have been collected at high altitude (supplementary table S1, Supplementary Material online).

To systematically inspect the function of the set of genes with signatures of positive selection, we further performed a gene ontology enrichment test using the Panther classification system (Mi et al. 2013). Gene ontology categories related to phosphorus metabolism and phosphorylation as well as more general metabolic and protein modification processes were enriched in genes with signatures of positive selection (supplementary table S3, Supplementary Material online). Enrichment of categories related to phosphorus metabolism are interesting given the low levels of available phosphorous in volcanic soils (Shoji et al. 1993) such as those found in Madeira.

### Madeirans Help Clarify Mainland Demography

The Madeiran population represents a relictual lineage that has been completely separated from continental populations for tens of thousands of generations. This pure relict lineage could be useful for reconstructing ancient Eurasian demography, which is complicated due to historical population expansions and admixture. In particular, since the Madeirans are close relatives of the Iberian relict lineage, we reasoned that they could illuminate the history and origin of this clade, which has been the focus of several recent studies (1001 Genomes Consortium 2016; Lee et al. 2017).

**Fig. 6.**
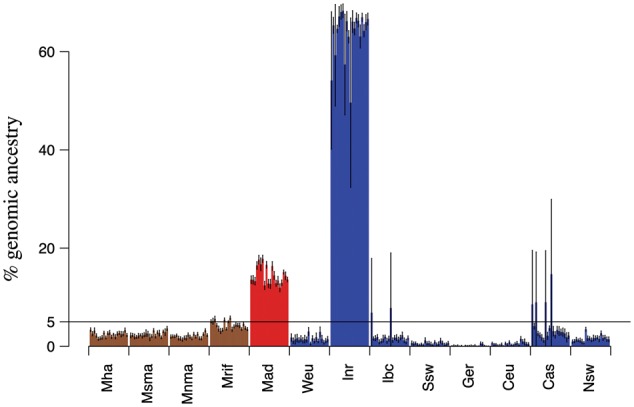
ChromoPainter analysis of Iberian relicts. Madeira as a potential donor represents the ancestral population that gave rise to the Iberian relicts as well. Columns represent the percentage of Iberian relicts’ genomes that first coalesces with members of each potential donor group. Eurasian groups are colored in blue, Madeira in red, and African groups in brown. The IDs for all mainland clades are described in figure 1. Vertical lines represent confidence intervals (± 1.96 · SE) across replicated analysis.

We used ChromoPainter to ask which present-day clusters best represent the parent population(s) of the Iberian relicts. Given that the Iberian relicts appear to best represent the parent population to the native Madeirans both based on genome-wide distance (fig. 1*c* and *d*) and on haplotype sharing (fig. 2*a*), we expected that the Madeirans would also represent the closest relatives to the Iberian relicts at the haplotype level. Instead, we found that the majority of Iberian relicts’ haplotypes find their closest relatives in the Iberian nonrelicts (fig. 6). This implies that the genomes of Iberian relicts are actually a mosaic of admixed relict and nonrelict haplotypes. This massive signature of admixture with nonrelicts across all Iberian relict individuals is in stark contrast to expectations based on previous studies (1001 Genomes Consortium 2016; Lee et al. 2017).

To better understand the history of admixture in the Iberian relicts, we used the *CCR* statistic based on eight haplotypes to compare Iberian relicts to the other mainland clades. The *CCR* statistic starts decaying from one toward zero around the time of the split between groups, but in several comparisons the trend reverts and *CCR* again increases toward one at 20–40 ka, suggesting secondary contact occurred at this time (supplementary fig. S10, Supplementary Material online). In particular, the Iberian relicts show signals of secondary contact with Iberian nonrelicts, Central Europe, Italy-Balkan-Caucasus, South Sweden, and partially with Western Europe, but not with Africa, Central Asia, North Sweden, or Germany. Further support for this time estimate comes from the finding that *N_e_* in Iberian relicts spikes at around the same time, consistent with an admixture event (fig. 3, and see fig. 5b in Durvasula et al. 2017).

**Fig. 7.**
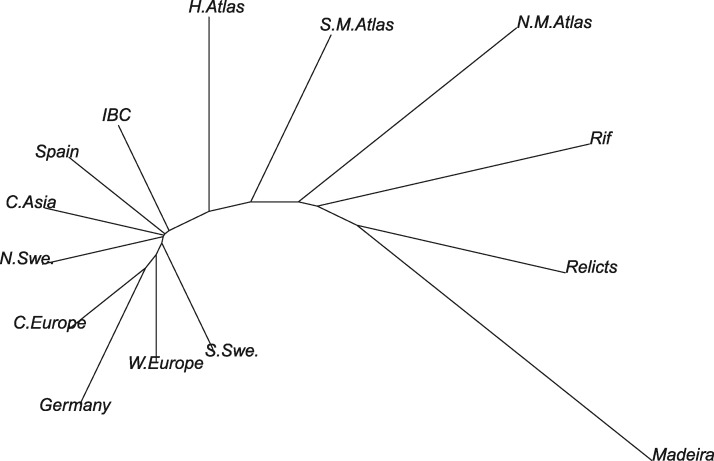
Relationships among the 13 mainland clades and Madeira. Neighbor joining tree of split time distances inferred by *CCR* (table 1).

The finding of massive admixture in the Iberian relicts raises new questions about their origin. Indeed, based on *CCR*, all Eurasian groups are more recently related to Iberian relicts compared with Africans (table 1 and supplementary fig. S10, Supplementary Material online), which may be due to the confounding factor of admixture. Using Madeirans as representatives of the ancestral clade that gave origin also to the original population of Iberians, the scenario for this second group drastically changes. Madeiran genomes, devoid of admixture signals, capture the original relationships of Iberian relicts, that are now closest to African clades, rather than to Eurasians (fig. 7 and table 1), supporting an African origin for this group.

**Table 1.** Matrix of Split Times among Madeira and the Nine Eurasian and Four Moroccan Clusters, Based on MSMC-*CCR*.

	Mad	Ir	Weu	Ceu	Ger	Ibc	Nsw	Ssw	Spa	Cas	Mnma	Msma	Mrif	Mha
Mad	0.0	72.8	85.6	93.8	91.6	88.9	86.7	88.5	89.0	86.8	86.7	88.4	79.8	87.6
Ir	72.8	0.0	63.3	71.2	68.9	62.9	63.5	63.7	64.4	62.1	76.8	73.0	72.6	70.9
Weu	85.6	63.3	0.0	30.6	30.6	30.6	30.6	30.6	30.6	30.6	69.3	50.1	72.1	46.9
Ceu	93.8	71.2	30.6	0.0	30.6	30.6	30.6	30.6	30.6	30.6	75.6	51.5	74.9	46.8
Ger	91.6	68.9	30.6	30.6	0.0	37.1	40.2	34.1	42.8	40.1	70.8	58.8	72.1	54.9
Ibc	88.9	62.9	30.6	30.6	37.1	0.0	30.6	30.6	30.6	30.6	65.7	47.3	69.9	43.6
Nsw	86.7	63.6	30.6	30.6	40.2	30.6	0.0	30.6	30.6	30.6	68.1	50.2	68.9	46.4
Ssw	88.5	63.7	30.6	30.6	34.1	30.6	30.6	0.0	30.6	30.6	71.8	49.8	71.6	46.2
Spa	89.0	64.4	30.6	30.6	42.8	30.6	30.6	30.6	0.0	30.6	67.6	48.4	69.9	44.5
Cas	86.8	60.0	30.6	30.6	40.1	30.6	30.6	30.6	30.6	0.0	68.8	47.7	70.0	44.9
Mnma	86.7	76.8	69.3	75.6	70.8	65.7	68.1	71.8	67.6	68.8	0.0	57.7	68.0	63.9
Msma	88.4	73.1	50.1	51.5	58.8	47.3	50.2	49.8	48.4	47.7	57.7	0.0	68.3	55.6
Mrif	79.8	72.6	72.1	74.9	72.2	69.9	68.8	71.6	70.0	69.9	68.0	68.3	0.0	68.7
Mha	87.6	70.9	46.9	46.8	54.9	43.6	46.4	46.2	44.5	44.9	63.9	55.6	68.7	0.0

These findings suggest that *A. thaliana* entered Eurasia both from the east, through the Middle East, and from the west, through Gibraltar. To further explore this hypothesis, we inferred the time to the split of all combinations of the nine Eurasian and four African main clusters with the *CCR* framework (table 1). A graph based on these *CCR* results showed that Iberian nonrelicts split relatively recently from Africans (fig. 7) compared with other Eurasian nonrelicts (second only to Italy-Balcans-Caucasus), providing further support for a second migration route through Gibraltar.

## Discussion

### Demographic and Adaptive History on Madeira

The study of a model organism in island conditions leads to several novel insights concerning long-distance dispersal events, and long-term effective population size. Our results imply that *A. thaliana* colonized Madeira in a single time window between 70 and 85 ka, with no further contacts between native Madeirans and any other continental clade. The colonization of Madeira clearly represents a natural event not mediated by humans, who had only just started populating Eurasia during this period (Timmermann and Friedrich 2016). In a species usually thought to evolve and spread as a human commensal (both in Eurasia [1001 Genomes Consortium 2016; Lee et al. 2017] and in North America [Platt et al. 2010]), the colonization of Madeira stands out as a clear example of a natural long-range dispersal event.

The closest mainland relatives to Madeira are the “Iberian relicts,” the most diverged cluster found in Eurasia (1001 Genomes Consortium 2016). Surprisingly, we find no evidence of a strong bottleneck during the colonization of Madeira. Specifically, the strongest bottleneck consistent with data had severity 0.1 (sensu Tajima 1989), which in a single long-distance dispersal event would correspond to a minimum of ten successful founders. Our results are consistent with a propagule dispersal model, where multiple individuals disperse together to colonize a new habitat. This could correspond either to a single dispersal event, where several seeds traveled together to the island, or to an ecological window of opportunity that allowed immigration and establishment of colonies over some short time interval. The absence of a signature of a strong bottleneck, together with the relatively high diversity in Madeira (4.3-fold lower than worldwide), are in line with the recent finding that island clades usually do not suffer from a marked reduction in *N_e_*, except shortly after a strong bottleneck (James et al. 2016). On the other hand, we find a clear signal of lower carrying capacity on the island compared with mainland, where long-term *N_e_* in Madeira stabilizes at around *N_e_* = 30 K. Nonetheless, the difference in diversity between Madeirans and their closest mainland relatives, the Iberian relicts, is only ∼3-fold, much smaller than expected based on differences in the geographic range. Consistent with an influence of the last glacial maximum on *A. thaliana* in Madeira, *N_e_* drops to 10 K 40 ka, and recovers to carrying capacity ∼15 ka.

The Madeiran clade is more uniform and isolated compared with mainland groups. This condition, unusual in *A. thaliana*, provides the opportunity to study adaptive processes at the local scale, without the confounding effects of admixture and secondary contact. The signature of a selective sweep on chromosome 1 in Madeirans overlaps with the major sweep signal in Eurasia (Clark et al. 2007; Long et al. 2013). However, the previously detected signature was for a derived translocation in this region. Interestingly, Madeiran samples carry the ancestral, non translocated variant, representing the first reported evidence of selection on the ancestral form of this translocation. If selection shaped patterns of variation at this locus, it did so independently for the widespread Eurasian variant, and for the ancestral one in Madeira, representing separate events of adaptation at the same locus.

Since the split between Madeiran *A. thaliana* and the Iberian relicts is ancient with respect to local effective population size (∼2.5 *N_e_* generations ago), adaptation events at colonization or during the establishment phase on the island are not detectable by SFS based scans for selective sweeps. Conversely, comparative approaches may capture signatures of early adaptations to the island conditions. Indeed, a McDonald–Kreitman test (McDonald and Kreitman 1991) reveals candidate genes with signatures of positive selection related to the response to pathogens and light, and to phosphorous metabolism. The signal related to the response to pathogens and light may underly adaptations to altitude in native Madeirans, as suggested for natural populations of *A. thaliana* in the North Italian Alps (Gunther et al. 2016). Indeed, all native Madeirans were found at high altitude (1,200–1,812 m). Further, volcanic soils like those in Madeira are known to be poor in available phosphorous (Shoji et al. 1993), and this characteristic, likely divergent from the source habitat before colonization, could have driven early adaptation to the local conditions.

### Madeira Informs Demography on the Continent

Native Madeirans are an archaic population, comprising variation that was represented on the continents in the past, but is today muddled by admixture. Therefore, they also help illuminate ancient demographic events in Eurasia. First, they show that the Iberian relict clade is actually the product of an ancient admixture event between two diverged lineages, one that potentially migrated from Africa via Gibraltar and the other from the Caucasus. Indeed the Iberian relicts do not constitute a pure relict clade, which would present equal divergence to the worldwide set as Madeirans, but rather a mosaic of admixed relict and nonrelict haplotypes. Second, our results imply that the westward spread of the now dominant Caucasian *A. thaliana* lineage occurred at least 20 ka, but likely much earlier.

These findings are in contrast to previous inferences. First, the westward expansion of Caucasian *A. thaliana* was thought to have occurred in the last ∼8 ka, spurred by the spread of agriculture (1001 Genomes Consortium 2016). Second, Lee et al. (2017) interpreted variation in Eurasia as consisting of three different clades. The Caucasian nonrelicts and the Iberian relicts were assumed to be pure groups, and the Iberian nonrelicts to result from admixture between the first two clades. This would have corresponded to a two-deme model with migration only from the minor clade into the major clade. Although this scenario is unlikely, given the higher prevalence of Caucasian nonrelicts in Eurasia, a good alternative was missing, in the absence of the Madeiran data representing the nonadmixed clade.

Our estimates of the time of admixture will need revision if common assumptions on mutation rate and generation time prove inappropriate for wild populations. However, we note that the mutation rate inferred from a natural population ($\mu =2.2\cdot 10^{-9}$ mutations per base pair per generation; Moises Exposito-Alonso, Claude Becker, Verena J. Schuenemann, Ella Reiter, Claudia Setzer, Radka Slovak, Benjamin Brachi, Joerg Hagmann, Dominik G. Grimm, Chen Jiahui, Wolfgang Busch, Joy Bergelson, Rob W. Ness, Johannes Krause, Hernan A. Burbano, Detlef Weigel, unpublished data) is on an average 3.3-fold lower than in a mutation accumulation experiment ($\mu =7.1\cdot 10^{-9}$ mutations per base pair per generation; Ossowski et al. 2010), likely in part due to purifying and background selection in nature. Therefore, our time estimates are more likely to be too recent rather than too ancient, and the mismatch with previous estimates, greater rather than smaller.

Madeiran genomes further help clarify the origin of Iberian relicts, a clade that has been the major focus of several studies (1001 Genomes Consortium 2016; Lee et al. 2017). The massive admixture signal, in fact, confounds any ancient relatedness, producing a pattern where Iberian relicts appear to split more recently from Eurasian, rather than African clades (supplementary fig. S10, Supplementary Material online). On the other hand, Madeiran samples reshape the species-wide phylogeny, revealing closer relatedness to African compared with Eurasian clades (figs. 4 and 7;table 1). Consistent with this view, gene genealogies in the native Madeiran samples show a much greater background signal of relatedness to African rather than Eurasian clades, likely in part due to incomplete lineage sorting (fig. 2*a*). These relationships among groups suggest an African origin of the common ancestor of Madeirans and Iberian relicts, as well as a possible second route of migration out-of-Africa through the narrow Gibraltar strait.

## Materials and Methods

### Samples, Alignment, and SNP-Calling

We sequenced the genomes of 14 accessions from the island of Madeira (fig. 1*a* and supplementary table S1, Supplementary Material online, European Variation Archive accession number PRJEB23751). We stratified seeds for 4 days, and we sequenced leaf material from plants grown in growth chambers in standard conditions by the Campus Support Facilities at the Campus Vienna Biocenter. We extracted DNA from young leaves using ThermoScientific GeneJet Plant Genomic DNA kits, quantified DNA content using a Qubit, and assessed quality with a Nanodrop machine. We prepared sequencing libraries with Illumina TruSeq DNA sample prep kits (Illumina, San Diego, CA) and we sequenced them on Illumina Hi-Seq instruments.

For alignment to the Arabidopsis TAIR10 reference genome and variant calling, we used the two pipelines described in Durvasula et al. (2017), with exactly the same parameters, settings, and software versions. The resulting average coverage varied between 20.5× and 33.2× across samples (supplementary table S1, Supplementary Material online).

### Origin of Madeiran *A. thaliana*

To overcome biases due to sample size differences among groups, in analyses of population structure (NJ, PCA, ChromoPainter), we evenly subsampled the nine Eurasian clusters identified in 1001 Genomes Consortium (2016), and the four Moroccan clusters identified in Durvasula et al. (2017) to 20 samples. Exceptions were only two clusters of Moroccan plants (Rif and North Middle Atlas), where fewer than 20 samples were available. We computed a whole genome neighbor joining tree using the R package ape v3.5 (Paradis et al. 2004), and a distance matrix based on pairwise differences per base pair, used also for the section on pairwise differences. The principal component analyses was performed using the pca option in PLINK (Purcell et al. 2007), after pruning for short-range linkage disequilibrium (indep-pairwise 50 10 0.1 option), and for missing data (geno 0 option). For ChromoPainter analyses (Lawson et al. 2012) on Madeiran samples, we randomly subsampled each of the 13 mainland clusters to 20 samples, 100 times independently to obtain even sample sizes across donor populations, and still represent the full diversity in the data set. For each subsampled set, we performed ten replicate runs of ChromoPainter, for a total of 1,000 replicated analyses. All recipient samples were analyzed independently. To estimate ancestry in the Iberian relicts, we used the same scheme of subsampling and iterates as for Madeirans, with the exception that the Iberian relicts were not used as a potential donor, but replaced by Madeirans as representatives of the ancestral population that gave rise to both groups.

### Demographic History of *A. thaliana* in Madeira MSMC

We inferred effective population size as a function of time $N_{e}(t)$ and split times between populations with the multiple sequentially Marcovian coalescent, MSMC v2 (Schiffels and Durbin 2014), and the *CCR* statistic. We used both the two-haplotype implementation, which is expected to produce unbiased results at older times (∼1.6 Ma–30 ka for our system) and the eight-haplotype configuration for better resolution of the recent past (as recent as 3 ka). The method cannot resolve very young population demography because of the low probability for coalescence events much more recent than the expected time to the first coalescence event, or ∼3 ka in Madeira, for the eight-haplotype implementation. Conversely, the method cannot resolve very old demographic events, because once most haplotypes reach their most recent common ancestor, genomes exhaust the information on rates of coalescence. This limitation is more marked when effective population sizes are low in the recent past, so coalescence rates are high, as in Madeira (this is clear from simulated data using the inferred Madeiran demography see supplementary figs. S6–S8, Supplementary Material online). Therefore *N_e_* for Madeiran samples is shown in figure 3 starting at 300 ka. For results across the full range of time intervals, see supplementary figure S2, Supplementary Material online.

For the inference of split times between Madeira and the mainland clusters, we calculated the cross coalescence rate (*CCR*), defined as the ratio of coalescence rates between groups to the average coalescence rate within groups across time intervals. *CCR* = 0 indicates a complete split, whereas *CCR* = 1 indicates that samples from the different clades are merged into a single group.

As usual for inbreeding organisms, we combined two inbred accessions, approximated as haploid genomes, into so-called “fake diploids.” For the inference of split times with the cross coalescence rate statistic (*CCR*), we combined the 11 native Madeiran samples into six pairs of fake diploids (using one sample twice), and the 18 Iberian relicts in nine pairs, plus the two Moroccan Zin samples into a single pair. We separately computed *CCR* over time for all possible combinations of one fake diploid from Madeira and one Iberian relict, then repeated the analyses comparing Madeira to the other 12 mainland clusters. To obtain estimates in real time, we assumed a generation time of 1 year, and a mutation rate inferred from mutation accumulation experiments (Ossowski et al. 2010).

For the inference of split times among continental groups, we used the same *CCR* threshold as for Madeira. To visualize relationships among continental clades, we used the neighbor joining algorithm on a matrix of split time distances (table 1).

### Simulations

All simulations used for the inference of the demographic history, and for inferences of selection, were performed with the coalescent simulator msprime (Kelleher et al. 2016), and all scenarios were replicated 200 times. In simulations, we used full genome size (five chromosomes, and real chromosome sizes), sample sizes as in real data (11 native Madeirans), mutation rates from mutation accumulation experiments (Ossowski et al. 2010), and a recombination map obtained from crosses (Salome et al. 2012) corrected for the rate of outcrossing events in natural populations (Bomblies et al. 2010).

In order to examine specific features of our demographic inference, we used a simulation scheme. Specifically, our approach consisted of simulating data under a range of demographic models and then inferring effective population size as a function of time, $N_{e}(t)$, using MSMC on the simulated data. Next, we used the output of MSMC as a (heuristic) summary statistic, comparing the estimated $N_{e}(t)$ from the real data with the $N_{e}(t)$ from simulation data.

As a baseline model, we used a demography that closely matches the $N_{e}(t)$ inferred from real data, with no additional population structure. Between present and 200 ka, we used the MSMC inference from Madeirans in eight-haplotype mode. For times more ancient than 200 ka, we used the inference from Iberian relicts, representing the parent population of Madeirans, in two-haplotype mode (details in the Supplementary Material online). For our model, we fixed *N_e_* to the *N_e_* estimate from the data at 23 time points evenly spaced on a logarithmic scale between 3 ka and 2 Ma. We then connected contiguous fitted points with an exponential function (supplementary fig. S5, Supplementary Material online). The time points used were chosen dense enough to ensure a good fit between the inferred $N_{e}(t)$ from real data and the $N_{e}(t)$ of the baseline model. $N_{e}(t)$ estimates for simulated data under the baseline model closely reproduced $N_{e}(t)$ estimates from real data. Building on this baseline model, we added variations to the model $N_{e}(t)$ used in simulations to check specific features of the demography (e.g., bottlenecks, timing of the split). Comparisons between inferred $N_{e}(t)$ on simulated and real data give us a measure of how different a model can be from the MSMC inference, and still produce results consistent with the observed data.

To find a threshold for the *CCR* statistic appropriate for Madeiran *A. thaliana*, we simulated data with the baseline demography, varying split times between 37 and 200 ka (supplementary fig. S3, Supplementary Material online). Then we computed *CCR* on simulated data in the same way as with real data. The *CCR* values overlapping the real split time in simulations varied between 0.70 and 0.75, deviating from results based on simulations with constant population size (*CCR* = 0.5; Schiffels and Durbin 2014). Moreover, we find that the more recent the split, the steeper the decay in *CCR*, and the higher the *CCR* value that crosses the real split time. Overall, in the time frame appropriate for Madeiran *A. thaliana*, the *CCR* statistic crosses the real split time when 0.70 <*CCR* <0.75, if all other aspects of the demography and biology of the species are well represented in the modeling scheme.

To explore the hypothesis of two rounds of migration to Madeira, we simulated data following the baseline model for $N_{e}(t)$, and letting 50% of Madeiran lineages merge with the continental group at the time of the more recent migration event, and the remaining at the time of colonization. The time of colonization was set to 85.4 ka, which among the first set of simulations best approximated the time at which *CCR* on real data started decaying from one. The time of the secondary migration event was let vary between 64.0 and 36.6 ka.

Simulations with variable mutation rates were performed again following the baseline model for $N_{e}(t)$, and allowing mutation rate to vary between estimates based on mutation accumulation experiments ($\mu =7.1\cdot 10^{-9}$ mutations per base pair per generation; Ossowski et al. 2010), and estimates based on natural expanding populations ($\mu =2.2\cdot 10^{-9}$ mutations per base pair per generation; Moises Exposito-Alonso, Claude Becker, Verena J. Schuenemann, Ella Reiter, Claudia Setzer, Radka Slovak, Benjamin Brachi, Joerg Hagmann, Dominik G. Grimm, Chen Jiahui, Wolfgang Busch, Joy Bergelson, Rob W. Ness, Johannes Krause, Hernan A. Burbano, Detlef Weigel, unpublished data). Specifically, each of the five chromosomes of *A. thaliana* was assigned a mutation rate so as to span this range.

To check whether a colonization bottleneck is visible in the MSMC framework, we simulated data following the baseline demography, and adding a bottleneck of varying severity (0.0 <severity <1.0) at the split time. Then we inferred $N_{e}(t)$ on simulated data with the same procedure as with real data (supplementary fig. S6*a*, Supplementary Material online). We also explored the possibility that a recent bottleneck (between 40 and 15 ka) would mask the effect of a more ancient one (at colonization), adding both bottlenecks to the baseline demography in simulations (supplementary fig. S6*b*, Supplementary Material online).

To understand the dynamics that produced the long period with low *N_e_* between 40 and 15 ka, we simulated data assuming a constant carrying capacity in Madeira (*N_e_* = 30 K) with a bottleneck in this time frame of varying duration and severity. In particular, duration was let vary between 10 and 30.000 generations, and severity between 0.1 and 2.0. For long bottlenecks, we simulated both an instantaneous, and a smooth recovery (supplementary fig. S8, Supplementary Material online).

### Inferences of Selection

To scan native Madeiran genomes for signatures of selection, we used SweepFinder2 (DeGiorgio et al. 2016), with grid sizes 5, 10, 50, and 100 kb and a recombination map estimated from crosses (Salome et al. 2012), corrected for the outcrossing rate in natural environments (Bomblies et al. 2010). Since results were coherent across grid sizes, we show just grid size 10 kb in the main figure.

For this analysis, we used both the original configuration, using information on polymorphic sites only, and the new configuration using also fixed derived variants (DeGiorgio et al. 2016). Since Madeira evolved in isolation from mainland clades, we polarized the site frequency spectrum to the reference genome, Col-0. The null distribution of the CLR statistic was determined with coalescent simulations following the six demographic models that best matched real data (supplementary fig. S8, Supplementary Material online). In particular, we simulated data following the baseline model, as well as following a model where Madeiran carrying capacity was constant and equal to 30 K from the split until present, with the exception of the last glacial maximum between 40 and 15 ka, where *N_e_* = 10 K, both with an instantaneous, or a smooth recovery to carrying capacity. For all scenarios, we simulated data with and without a colonization bottleneck of severity 0.1 at the split from Iberian relicts, the strongest bottleneck still consistent with observed data. For each of these six scenarios, we computed the CLR statistic with SweepFinder2 on simulated data. Across demographic scenarios, the highest 99th percentile of the CLR distribution was used as a significance threshold to reject the null model of neutrality.

Although the peak in *CLR* on chromosome 1 was significant in both sweepFinder2 configurations, three other peaks were significant only when using both polymorphisms and fixed derived variants (supplementary fig. S9, Supplementary Material online). Since Madeirans are very diverged from the reference genome at the nucleotide level, there are likely also many structural differences. Misaligned reads, especially at the breakpoints of structural variants could result in excess fixed derived differences from the reference genome, and therefore produce false positive sweep signatures. Indeed, we found rearrangements nearby all the significant CLR peaks. However, we do not expect similar spurious signatures at polymorphic sites, therefore, we focussed our discussion of selection on signals that overlapped in both sweepFinder2 configurations.

For the McDonald–Kreitman test, we annotated genomic variants in Madeirans and Iberian relicts with snpEff (Cingolani et al. 2012). Assessment of significance on the contingency table was done using a Fisher’s exact test implemented in R (fisher.test() function). Gene ontology enrichment tests were performed using the Panther classification system (Mi et al. 2013). No signal was significant after Bonferroni correction for multiple hypothesis testing. 

## Supplementary Material

Supplementary data are available at *Molecular Biology and Evolution* online.

## Supplementary Material

Supplementary Data
