# Supplementary material for: Madeiran Arabidopsis thaliana Reveals Ancient Long-Range Colonization and Clarifies Demography in Eurasia
Source: Mol Biol Evol. 2017 Dec 5;35(3):564–74. doi: 10.1093/molbev/msx300 (PMC5850838; doi:10.1093/molbev/msx300)
Supplement: Supplementary Data [file msx300_supp.pdf]

Supplementary Material
Madeiran *Arabidopsis thaliana* reveals ancient long-range colonization and clarifies demography in Eurasia

Andrea Fulgione[1,2,*], Maarten Koornneef[3,4], Fabrice Roux[5], Joachim Hermisson[1,6], and Angela Hancock[1,3,*]

[1]*Max F. Perutz Laboratories, University of Vienna, 1030 Vienna, Austria,* [2]*Vienna Graduate School of Population Genetics, Vienna, Austria,* [3]*Max Planck Institute for Plant Breeding Research, 50829 Cologne, Germany,* [4]*Wageningen University, NL-6708 PE, Wageningen, The Netherlands,* [5]*LIPM, Université de Toulouse, INRA, CNRS, Castanet-Tolosan, France,* [6]*Department of Mathematics, University of Vienna, 1090 Vienna, Austria,* [*]*To whom correspondence should be addressed*

Supplementary methods

δaδi

For a test of our demographic inference, we used the composite likelihood approach, δaδi (Gutenkunst et al. 2009). To mitigate the confounding effect of sampling and population structure in the joint site frequency spectrum of the Madeiran clade, we eliminated nearly identical samples (one for each pair with 10^3 times lower pairwise differences per base pair, compared to average comparisons within Madeira), as well as single samples collected in isolated parts of the island, which accounted for the majority of the signal of, respectively, excess doubletons and singletons. We based the analyses on the joint site frequency spectrum computed on intergenic sites only, assuming they should evolve mostly neutrally. We replicated the analyses 200 times independently for each demographic model, with different, randomly chosen, starting values for each parameter within predefined ranges, and with a maximum number of iterations of 50.

We modelled a number of possible demographic histories of increasing complexity. Specifically, in all models the Madeiran clade splits from the Iberian relicts at some time T_{split}, followed by either constant population sizes within demes (the "Simple split" model in supplementary table S2), or allowing for exponential changes in $N_e(t)$ (the "Exp.growth" model). We also tested the possibility of a colonisation bottleneck in Madeira, both constraining it to happen at the split (the "Bot.split" model), or at any time between the split and present (the "Bot.free" model). In all models, the parameter boundaries for optimization was set to (10^{-3}; 20) for N_e, and (0; 10) for the split time. These ranges include, and are larger than those suggested in δaδi's manual (Gutenkunst et al. 2009). Optimised parameters for all models are shown in (supplementary table S2).

Supplementary text

MSMC

We explored the possibility of multiple colonisation events to Madeira through simulations. With a first ancient colonisation at 85.4 kya, and a second more recent round of migration at 64 or 48 kya, the decay in CCR extends from

the colonisation to the more recent migration event. However, both scenarios still produce a faster decay in CCR than in real data. Conversely, a second migration event as recent as 36.6 kya produced a marked spike in CCR, absent in real data. We also investigated whether the minimum in N_e between 10 and 40 kya may represent the colonisation of Madeira, rather than the ice age, smoothed across time in MSMC. Simulations with a sharp colonisation bottleneck in this time frame (supplementary fig. S7a), even with duration up to 500 years (supplementary fig. S7b) could not recover the broad signal in real data. Rather, a carrying capacity of around $N_e =$ 30K in Madeira, plus a relatively long period (between 40 and 15 kya, but not as long as between 40 and 10 kya) with lower N_e ($N_e = 10$K) possibly corresponding to the last glacial maximum is consistent with observed data, and a smooth recovery back to carrying capacity fits the data better than a sudden recovery (supplementary fig. S7b, and S8).

δaδi

All models produced reasonable estimates for the effective population size before the split, that was inferred to be between 120 and 140 K. Also the inferred time to the split was relatively constant across models, varying between 75 and 87 kya, and very closely agreeing with inferences based on MSMC. In the model with constant effective population sizes after the split, N_e in Madeira was optimised to 24K, and in the model allowing for exponential changes in $N_e(t)$ it decayed from around 50K to 19K, in both cases broadly agreeing with inferences from MSMC (long term N_e in Madeira fluctuating around 30K). Both bottleneck models had more parameters and a lower likelihood than the exponential model, so overall they were outperformed by simpler scenarios. When we constrained the bottleneck to happen at the split, the optimised N_e right after the split was actually greater than long-term effective population size in Madeira, consistent with results from MSMC, and inconsistently with a colonisation bottleneck at the split. When the bottleneck was not bound to happen at the split, the optimised timing and N_e (between 32 and 1 kya, N_e =14.7K) broadly coincided with the "ice age" period inferred by MSMC (between 40 and 15 kya, $N_e = 10$K).

The McDonald-Kreitman test

The McDonald-Kreitman test (McDonald and Kreitman 1991) resulted in 15 genes with signatures consistent with positive selection.

These genes were: AT1G14100, AT1G28450, AT1G31550, AT1G40129, AT1G65190, AT2G28960, AT3G29810, AT4G00960, AT4G17020, AT4G19050, AT4G21250, AT5G06540, AT5G11250, AT5G42310, AT5G43350.

References

DeGiorgio M, Huber CD, Hubisz MJ, Hellmann I, Nielsen R. 2016. SweepFinder2: increased sensitivity, robustness and flexibility. Bioinformatics 32:1895-1897.

Gutenkunst RN, Hernandez RD, Williamson SH, Bustamante CD. 2009. Inferring the joint demographic history of multiple populations from multidimensional SNP frequency data. PLoS Genet 5:e1000695.

McDonald JH, Kreitman M. 1991. Adaptive protein evolution at the Adh locus in Drosophila. Nature 351:652-654.

Supplementary figures

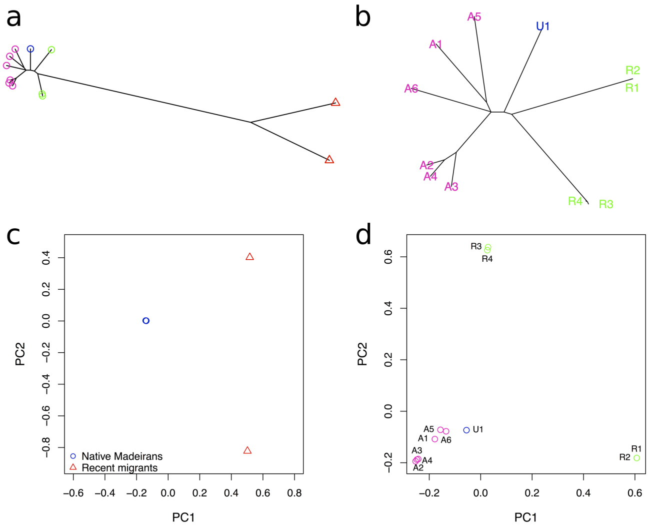

Figure S1: Population tructure in Madeira. (*a*, *b*) Neighbour joining tree of Madeirans, with and without the three recent migrants; (*c*, *d*) Principal component analysis of Madeirans, with and without the three recent migrants. Sample IDs are described in table S1. Triangles represent the three recent migrants (P1-3); circles represent native Madeirans (11 samples). Among native Madeirans, different colours represent different geographic regions (as in table S1). At the scale of subfigure *a* and *c*, two of the recent migrants are almost identical, and so are the native Madeirans in subfigure *c*.

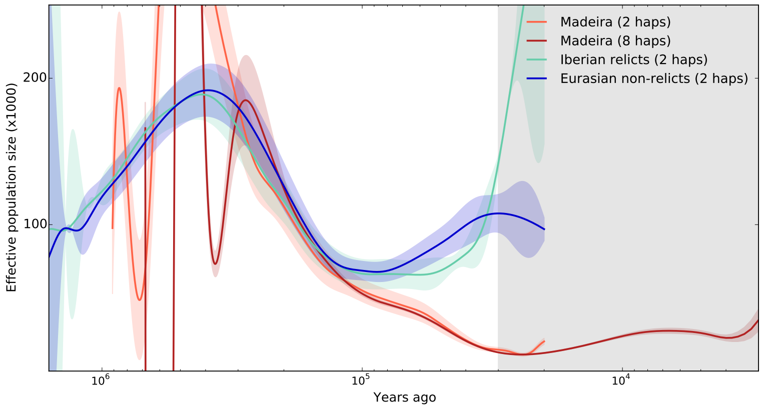

Figure S2: Effective population size as a function of time ($N_e(t)$) in Madeira (two shades of red), Iberian relicts (green) and Eurasians (blue). $N_e(t)$ is shown smoothed with a cubic spline across median N_e for each time segment used in MSMC. Shaded areas represent confidence intervals ($\pm$ 1.96 $\cdot$ SE). Due to small N_e in the recent past, Madeiran genomes exhaust earlier haplotype information, so MSMC inference is not anymore reliable for times more ancient than about 200 kya in 8-haplotypes mode, 300 kya in 2-haplotypes mode.

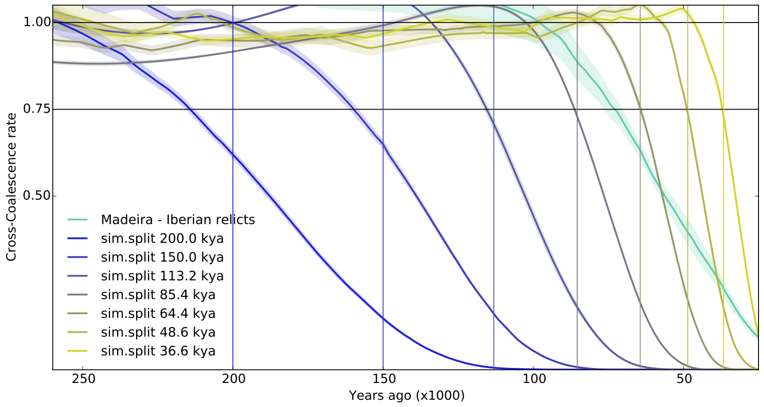

Figure S3: Simulations with varying split times. Simulations follow $N_e(t)$ inferred with MSMC for Madeirans, varying the time to the split from Iberian relicts between 37 and 200 kya. CCR over time was computed on simulated data, with the same procedure as with real data. The decay in CCR between Madeira and Iberian relicts is shown for comparison. Lines represent the median across 200 replicated simulations, the shaded areas confidence intervals ($\pm$ 1.96 $\cdot$ SE). Real split times in simulations are represented as vertical lines of the same color as the simulations they refer to.

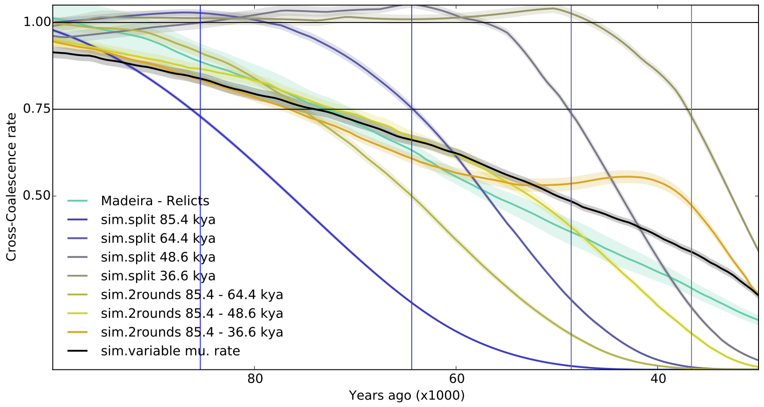

Figure S4: Two rounds of migration and variable mutation rate. CCR decay with two rounds of migration to the island (a first colonization at 85.4 kya and a second migration event at, respectively, 64.4, 48.6 and 36.6 kya), and CCR decay with increased variance in the mutational process. Simulations with instantaneous splits in the same time frame are shown for comparison, as well as real data on the split between Madeira and Iberian relicts.

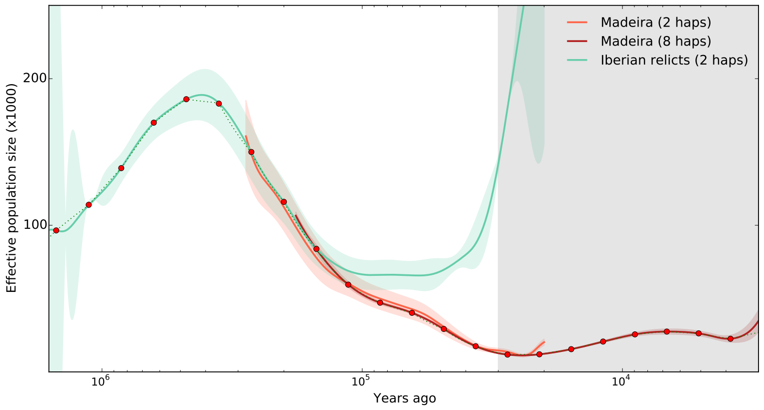

Figure S5: Baseline model for simulations. $N_e(t)$ inferred from Madeirans and Iberian relicts is used to create a baseline demographic model. Red dots represent 23 time points evenly spaced on a logaritmic scale between 3 kya and 2 Mya, at which we fixed simulated N_e to the value inferred from real data. Red dots were connected with exponential functions, represented as a dotted line.

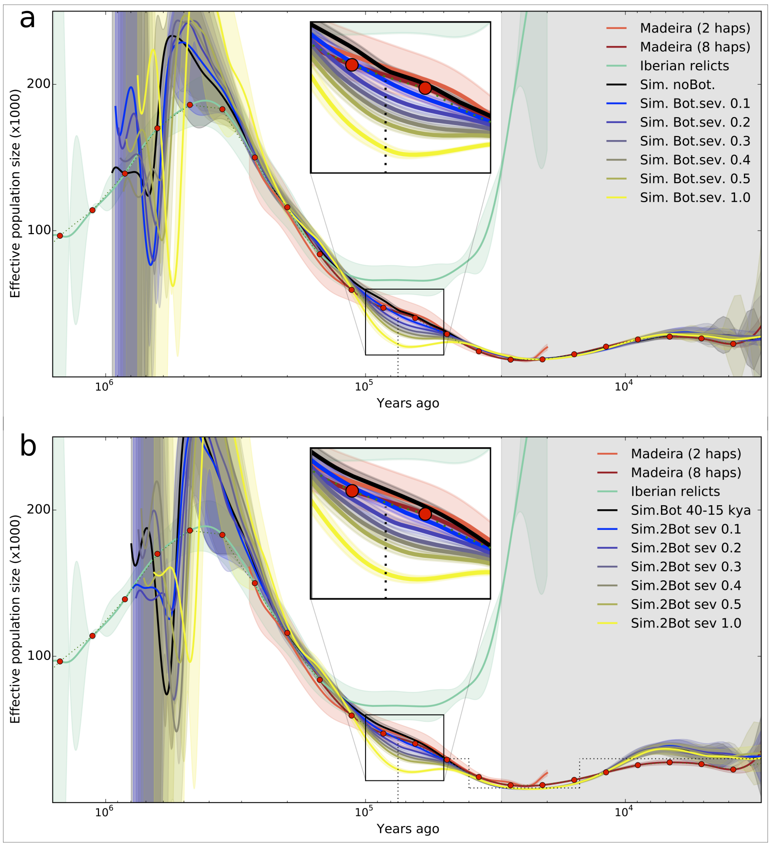

Figure S6: Simulated colonization bottleneck. (a) MSMC inference on simulated data adding to the baseline model a colonization bottleneck of varying severity (0.1, 0.2, 0.3, 0.4, 0.5, 1.0) at the split (75 kya). (b) MSMC inference on simulated data with a carrying capacity in Madeira of $N_e = 30K$, a long bottleneck corresponding to the last glacial maximum (40-15 kya, $N_e = 10K$), as well as a colonization bottleneck of varying severity. Dotted lines depict the corresponding simulated trajectories in $N_e(t)$. The inset in each sub-figure represents a zoom into the time frame under focus.

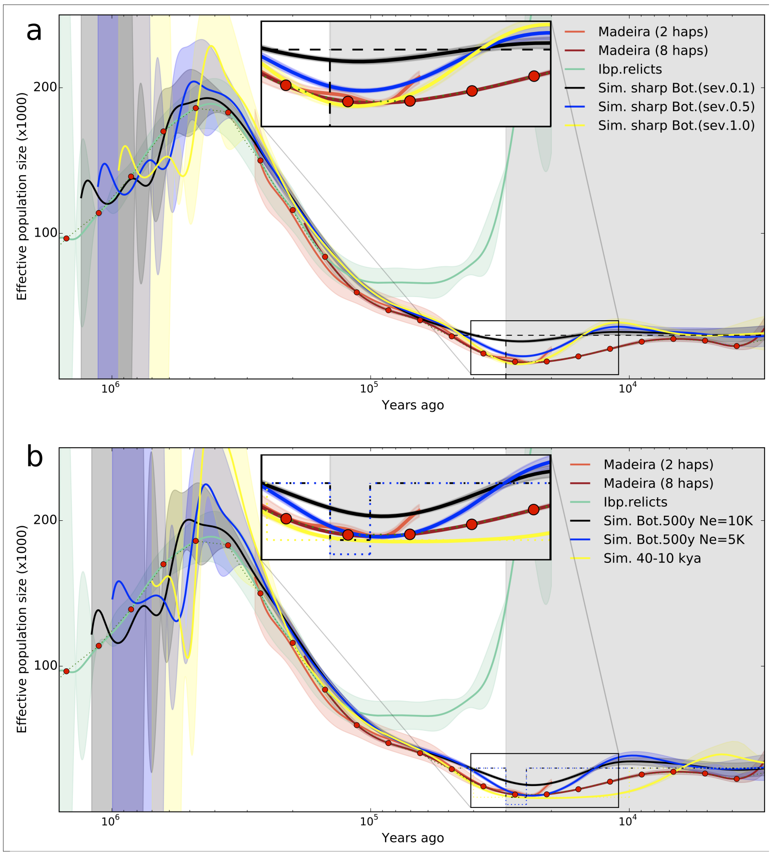

Figure S7: Simulated ice age bottleneck. (*a*) MSMC inference on simulated data with a carrying capacity in Madeira of N_e=30K, and a sharp bottleneck of varying severity at the last glacial maximum; (*b*) MSMC inference on simulated data with a long (between 500 and 30000 generations) bottleneck of varying severity at the last glacial maximum. Dashed lines depict the different simulated trajectories in $N_e(t)$, distinguished in subfigure *b* with colors corresponding to the simulated trajectory. The inset in each sub-figure represents a zoom into the time frame under focus.

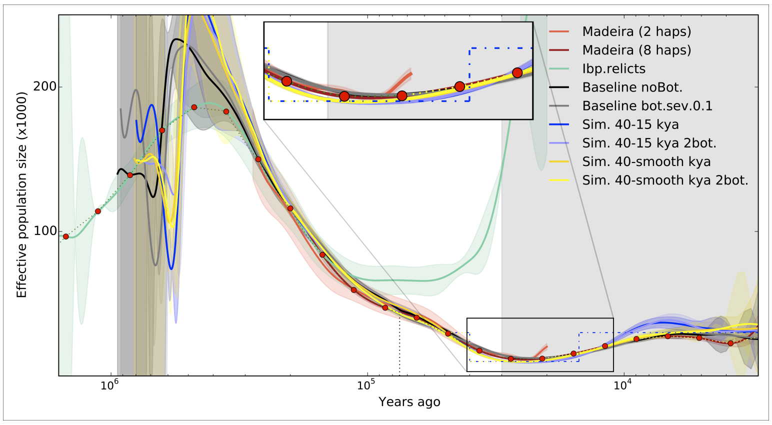

Figure S8: The six best fitting models. Three demographic models produced changes in $N_e(t)$ consistent with real data, with or without a colonization bottleneck of severity 0.1, for a total of six models. The first was the baseline model, that followed inferred $N_e(t)$ from MSMC. The second and third models assumed a carrying capacity in Madeira of $N_e = 30K$, and an ice age bottleneck (N_e=$10K$ between 40 and 15 kya) with respectively a sudden, and a smooth recovery to carrying capacity. Dashed and dotted lines of different colors depict the corresponding simulated trajectories in $N_e(t)$.

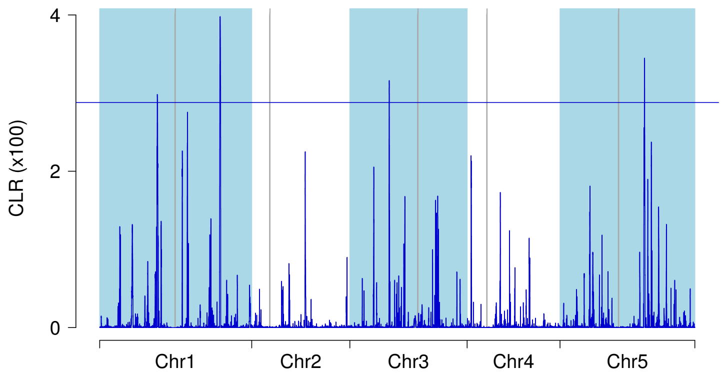

Figure S9: CLR in Madeiran *A. thaliana*. *CLR* statistic computed with SweepFinder2 (DeGiorgio et al. 2016), using polymorphic and fixed derived variants. The horizontal line represents a significance threshold obtained with simulations (see Materials and Methods).

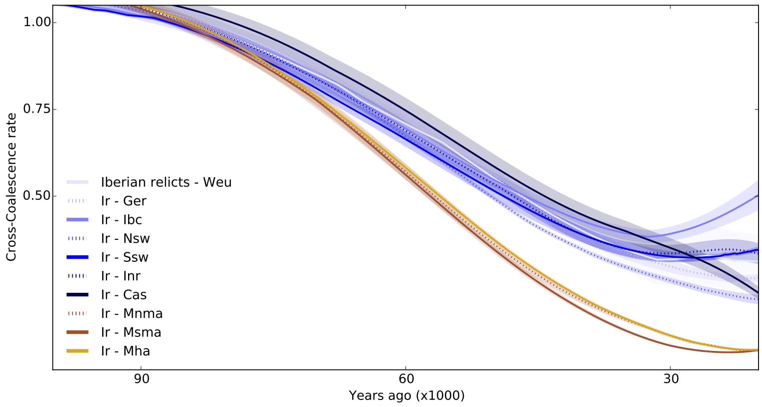

Figure S10: Changes over time in CCR (8-haplotypes mode) between Iberian relicts and the other mainland groups.

Table S1: Samples informations. Are: Pico do Areeiro; Pal: Palheiro; Rab: Rabacal; Rui: Pico Ruivo; M.K.: Maarten Koornneef; F.R.: Fabrice Roux; S.H.: Svante Holm; n.r.: nature reserve; p.g.: public gardens.

ID	Region	Latitude	Longitude	Altitude	Collector	Site info	Mean coverage
A1	Are	32.740	-16.930	1812	F.R.	n.r.	31.0
A2	Are	32.740	-16.930	1812	F.R.	n.r.	26.9
A3	Are	32.740	-16.930	1812	F.R.	n.r.	29.4
A4	Are	32.740	-16.930	1812	F.R.	n.r.	27.7
A5	Are	32.740	-16.930	1812	F.R.	n.r.	29.4
A6	Are	32.745	-16.937	1600	S.H.	n.r.	24.9
P1	Pal	32.658	-16.866	509	M.K.	p.g.	27.5
P2	Pal	32.659	-16.867	524	M.K.	p.g.	29.9
P3	Pal	32.659	-16.866	521	M.K.	p.g.	30.7
R1	Rab	32.754	-17.130	1200	M.K.	n.r.	25.0
R2	Rab	32.754	-17.130	1200	M.K.	n.r.	33.2
R3	Rab	32.754	-17.130	1200	M.K.	n.r.	26.4
R4	Rab	32.758	-17.132	1200	S.H.	n.r.	20.5
U1	Rui	32.759	-16.943	1800	S.H.	n.r.	22.0

Table S2: δaδi, parameter optimizations

Model name	par.	logLik.	T_{split}	N_0	N_1	N_2	N_3	N_4	T_{Bot1}	T_{Bot2}	N_{Bot}
Simple split	3	-3657.9	75.0	127	24	N_1	900	N_2	-	-	-
Exp.growth	4	-2952.4	86.8	141	50	19	91	2800	-	-	-
Bot.split	5	-3336.4	79.0	122	N_{Bot}	16	930	N_3	T_{split}	39	390
Bot.free	6	-3335.8	80.0	123	138	N_1	930	N_3	32	1	14

Table S3: GO enrichment test for genes with signatures of positive selection in a McDonald Kreitman test. Only enrichment for categories represented by more than a single gene are shown.

GO biological process	upload	fold Enrichment	p-value
organic substance metabolic process (GO:0071704)	8	2.14	1.68E-02
primary metabolic process (GO:0044238)	8	2.30	1.10E-02
metabolic process (GO:0008152)	8	1.82	4.42E-02
macromolecule metabolic process (GO:0043170)	7	2.65	9.09E-03
cellular macromolecule metabolic process (GO:0044260)	7	2.92	5.22E-03
cellular metabolic process (GO:0044237)	7	2.00	4.03E-02
protein modification process (GO:0036211)	5	5.78	1.17E-03
cellular protein modification process (GO:0006464)	5	5.78	1.17E-03
cellular protein metabolic process (GO:0044267)	5	4.14	5.13E-03
protein metabolic process (GO:0019538)	5	3.62	9.02E-03
organonitrogen compound metabolic process (GO:1901564)	5	2.74	2.78E-02
macromolecule modification (GO:0043412)	5	5.16	1.95E-03
phosphate-containing compound metabolic process (GO:0006796)	4	5.16	6.15E-03
phosphorus metabolic process (GO:0006793)	4	4.99	6.94E-03
phosphorylation (GO:0016310)	4	7.07	1.98E-03
protein phosphorylation (GO:0006468)	4	10.65	4.30E-04
polysaccharide biosynthetic process (GO:0000271)	2	25.15	2.82E-03
plant-type cell wall organization or biogenesis (GO:0071669)	2	20.26	4.30E-03
glucan biosynthetic process (GO:0009250)	2	46.16	8.54E-04
cell wall organization (GO:0071555)	2	9.19	1.95E-02
cell wall organization or biogenesis (GO:0071554)	2	7.03	3.21E-02
external encapsulating structure organization (GO:0045229)	2	8.68	2.17E-02
single-organism carbohydrate metabolic process (GO:0044723)	2	10.05	1.65E-02
cell wall biogenesis (GO:0042546)	2	22.37	3.54E-03
polysaccharide metabolic process (GO:0005976)	2	11.43	1.29E-02
carbohydrate biosynthetic process (GO:0016051)	2	15.59	7.14E-03
cellular carbohydrate biosynthetic process (GO:0034637)	2	23.23	3.29E-03
cellular glucan metabolic process (GO:0006073)	2	24.98	2.86E-03
cellular polysaccharide metabolic process (GO:0044264)	2	19.19	4.77E-03
cellular carbohydrate metabolic process (GO:0044262)	2	13.46	9.46E-03
plant-type cell wall biogenesis (GO:0009832)	2	34.08	1.55E-03
cell wall polysaccharide metabolic process (GO:0010383)	2	39.21	1.18E-03
glucan metabolic process (GO:0044042)	2	24.98	2.86E-03
cell wall macromolecule metabolic process (GO:0044036)	2	30.65	1.91E-03
cellular polysaccharide biosynthetic process (GO:0033692)	2	30.65	1.91E-03
